# Prediction of H3K27M alteration in midline gliomas of the brain using radiomics: A multi-institute study

**DOI:** 10.1093/noajnl/vdae153

**Published:** 2024-09-10

**Authors:** Abhilasha Indoria, Ankit Arora, Ajay Garg, Richa S Chauhan, Aparajita Chaturvedi, Manoj Kumar, Subhas Konar, Nishanth Sadashiva, Shilpa Rao, Jitender Saini

**Affiliations:** Neuroimaging and Interventional Radiology, National Institute of Mental Health and Neuro Sciences (NIMHANS), Bengaluru, India; Neuroimaging and Interventional Radiology, National Institute of Mental Health and Neuro Sciences (NIMHANS), Bengaluru, India; Neuroimaging and Interventional Neuroradiology, All India Institute of Medical Sciences, New Delhi, India; Radio-Diagnosis, All India Institute of Medical Sciences (AIIMS) Raipur, India; Neurosurgery, National Institute of Mental Health and Neuro Sciences (NIMHANS), Bengaluru, India; Neuroimaging and Interventional Radiology, National Institute of Mental Health and Neuro Sciences (NIMHANS), Bengaluru, India; Neurosurgery, National Institute of Mental Health and Neuro Sciences (NIMHANS), Bengaluru, India; Neurosurgery, National Institute of Mental Health and Neuro Sciences (NIMHANS), Bengaluru, India; Neuropathology, National Institute of Mental Health and Neuro Sciences (NIMHANS), Bangalore, India; Neuroimaging and Interventional Radiology, National Institute of Mental Health and Neuro Sciences (NIMHANS), Bengaluru, India

**Keywords:** diffuse midline gliomas, H3K27M altered, machine learning, radiomics

## Abstract

**Background:**

Noninvasive prediction of H3K27M-altered Diffuse midline gliomas is important because of the involvement of deep locations and proximity to eloquent structures. We aim to predict H3K27M alteration in midline gliomas using radiomics features of T2W images.

**Methods:**

Radiomics features extracted from 124 subjects (69 H3K27M-altered/55 H3K27M-wild type). T2W images were resampled to 1 × 1 × 1mm^3^ voxel size, preprocessed, and normalized for artifact correction, intensity variations. The feature set was normalized and subjected to reduction by variance thresholding, correlation coefficient thresholding, and sequential feature selector. Adaptive synthesis oversampling technique was used to oversample the training data. Random forest classifier (RFC), Decision tree classifier (DTC), and K-nearest neighbors classifier (KNN) were trained over the training dataset and the performance was assessed over the internal test dataset and external test data set (52 subjects: 33 H3K27M-altered/19-H3K27M-wild type).

**Results:**

DTC achieved a validation score of 77.33% (5-fold cross-validation) and an accuracy of 80.64%, 75% on internal and external test datasets. RFC achieved a validation score of 80.7% (5-fold cross-validation) an accuracy of 80.6%, and 73% on internal and external test datasets. DTC achieved a validation score of 78.67% (5-fold cross-validation) an accuracy of 80.64%, and 61.53% on internal and external test datasets. The accuracy score of DTC, RFC, and KNN on the internal test dataset was approximately 80% while on the external test dataset, DTC achieved 75% accuracy, RFC achieved 73% accuracy and KNN achieved 65.1% accuracy.

**Conclusions:**

H3K27M alteration is a potential immunotherapeutic marker and is associated with poor prognosis and radiomics features extracted from conventional T2W-images can help in identifying H3K27M-altered cases non-invasively with high precision.

**Key Points**

- Complete surgical resection or even biopsy may not always be feasible for tumors and midline structures because it involves deep structures and proximity to eloquent areas.
- Radiomics can capture complete lesion characteristics quantitatively and can be used in disease diagnosis.
- A radiomics-based machine learning model was developed to non-invasively predict H3K27M alteration for tumors at midline locations and was tested on internal as well as external datasets.

**Importance of Study**

H3K27M alteration is known to be associated with poor prognosis independent of age, tumor location, and histopathological grade. This alteration is also represented as a potential target for immunotherapy. However, identification of this mutation is dependent on biopsy and DMGs can involve deep structures where biopsy is not always possible. Noninvasive diagnosis from readily available conventional MRI images can therefore assist in the identification of H3K27M alteration. Radiomics is a quantification method that allows the characterization of complete lesion phenotype. The present study aimed to assess the value of conventional T2W MRI radiomics in tumors located in the midline structures by testing machine learning models on internal as well as external validation datasets. Results of this study demonstrate that radiomics has the potential to identify H3K27M altered gliomas with decent accuracy and precision.

Diffuse midline gliomas (DMG) are a distinct group of brain tumors, first introduced in the 2016 World Health Organization classification of central nervous system and its definition was further refined in the latest classification where they have been defined as tumors located in midline and are H3K27M-altered, to recognize different mechanisms altering the pathogenic pathway in these tumors.^1,2^ DMG can affect children as well as young adults and may involve the thalamus, pons, and other midline structures such as the midbrain, medulla, and spinal cord.^3–5^ DMGs have been reported to contribute approximately 15% of all childhood brain tumor-related deaths.^6^ About 1 in 5 childhood brain tumors and about 4 in 5 of all brainstem tumors fall into the DMG category.^6,7^ DMG in adults, has been estimated to constitute about 3% of infiltrating gliomas.^8^ H3K27M alteration can be harbored in either the histone H3F3A gene, which encodes the histone H3 variant H3.3, or HIST1H3B gene, which encodes the histone H3 variant H3.1. This alteration has been associated with poor prognosis independent of age, tumor location, and histopathologic grading^9,10^ and represents a potential target for immunotherapy.^11^ DMG can involve deep structures or can be proximal to eloquent structures, biopsy may not always be feasible in such cases. Noninvasive identification of this alteration can help in cases where biopsy is inconclusive or not feasible. Initial experience of identification of H3K27M wild type and altered tumors has been found to be difficult using qualitative assessment of magnetic resonance imaging (MRI).^12,13^

Radiomics involves the extraction and analysis of a large number of quantitative features from medical images using advanced computational methods. These features can capture lesion characteristics quantitatively and can be used in improving the accuracy of disease diagnosis, patient outcome, and guiding treatment decisions. Radiomics relies on the idea of capturing the spatial and intensity relationship between individual pixels by using statistical, structural, and spectral characteristics.^14,15^ Radiomics features can hence capture the subtle differences in the imaging characteristics of tumors that may not be apparent to the human eye and may be indicative of their biological behavior. Radiomics-machine learning (ML) based approaches have shown promise in oncologic imaging analysis and are pacing rapidly. There are many studies that have been conducted for the prediction of various molecular markers in tumors, for example, methylation of O(6)-methylguanine methyltransferase (MGMT), 1p19q codeletion, mutation in isocitrate dehydrogenase (IDH), and p53, with more than 80% accuracy.^16–18^ However, there are very few published studies trying to predict H3K27M alteration prediction using the radiomics approach.^19^ The lack of external validation datasets challenges the generalizability of radiomics.

With this background, the aim of this study is to develop an ML-based radiomics model using T2W MRI images to predict the H3K27M alteration status of midline gliomas on internal as well as external test datasets. T2W images were utilized on account of the easy availability of these images in all cases. Furthermore, we believed that tumor segmentation was more feasible on T2W images compared to the contrast-enhanced T1W images due to the poor demarcation of the tumor border on these images.

## Methods

### Patient Selection

Ethics was obtained from the institutional ethics committee and the requirement for informed consent was waived owing to the retrospective nature of the study. The neuropathology database of our institute was reviewed and cases with H3K27M alteration status were identified. Available preoperative MRI scans of identified subjects were retrieved from the Picture Archiving and Communication System. Cases were excluded from the study if: (1) preoperative MRI scans were not available, (2) if scans were of inadequate diagnostic quality, (3). The lesion was not located at the midline brain structures, (4) At the time of image acquisition, the patient has undergone any procedure such as surgery, biopsy, or chemoradiotherapy.

In the internal dataset, 124 cases were included, 55 cases with H3K27M wild-type status and 69 cases with H3K27M altered status. In the external validation dataset 81 subjects, out of which 52 cases fit into the inclusion criteria of this study (33 H3K27M altered and 19 H3K27M wild type), were included as the external test dataset.

### Immunostaining

All the cases are classified according to WHO 2016 criteria and were reviewed by the neuropathologist, histologically categorized as phenotypic low-grade diffuse astrocytomas (grade II), anaplastic astrocytomas (grade III), glioblastomas (grade IV), and DMGs (grade IV).

For immunohistochemistry (IHC), silane-coated slides were used to collect formalin-fixed paraffin-embedded sections (4 µm) from the blocks. The Ventana Benchmark automated staining system (Ventana Benchmark-XT) was used for IHC. Antigen retrieval was performed using the sections, afterwards, incubation with primary and then secondary antibodies was done. Hematoxylin was used for counterstaining. Following antibodies were used: H3K27me3 (Millipore, 07–449; 1:100; H3.3K27Mme3, Malaysia, RM192, 1:100); Anti-mIDH1 R132H (dilution 1:50, internal clone H06, Dianova, Hamburg, Germany); Sigma polyclonal anti ATRX antibody in 1:100 dilutions; and DAKO P53 antibody in 1:200 dilutions, D07 clone. Appropriate negative or positive controls were used for each batch of staining.

### MRI Acquisition

#### Internal dataset.—

All subjects underwent MRI scans using either 1.5 (*Aera 1.5 T, Siemens Medical Systems, Erlangen, Germany*) or 3.0 (*Ingenia 3T, Philips Medical Systems, Best, Netherlands*) Tesla MR Scanners. The 32-channel head coil was used as per standard operating procedure with/without sedation. From the standard MRI protocol, the following sequences were retrieved: T2-weighted Turbo spin-echo axial and coronal (Repetition Time-TR/ Echo Time-TE: 3000–4900/80–99 ms) and fluid-attenuated inversion recovery (FLAIR; TR/TE: 9000–11 000/87–125 ms, IR delay 2500–2800 ms). Radiomic features were extracted using T2W sequences. Different sequence parameters were recorded according to different clinical protocols.

#### External dataset.—

T2W images were provided by a different institute, all the scans were acquired using Ingenia 3T Philips scanner following their standard clinical protocol with TE: 248 ms, TR: 3500 ms, and Flip Angle: 90.

### Lesion Segmentation, Image Preprocessing, and Feature Extraction

**Figure 1.**
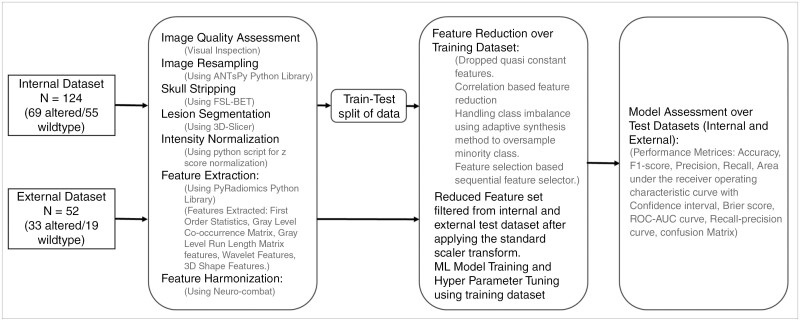
An overview of the flow of the entire study.

The general workflow of the analysis is provided in Figure 1. All images were resampled to 1X1X1 mm^3^ voxel size. Images were skull-stripped using the FSL brain extraction tool (FSL BET) through shell script. The whole tumor volumes were segmented by a neuroradiologist with 5 years of experience, blind to the H3K27M alteration status using an intensity threshold-based semi-automatic method of 3D slicer version 4.10.2. Since these are known to be infiltrative tumors, intratumoral necrotic, cystic changes as well as the peritumoral edema were included in segmentation therefore, the mask included the complete T2W abnormal signal. The example of the segmentation is provided in Supplementary Figure 1. All the segmented masks were validated by a neuroradiologist with more than 15 years of experience in an academic institute. Segmented 3D tumor volumes were saved as binary maps for the extraction of radiomics features. Intensity normalization using the z score was performed on the T2W images. An open-source Python library called PyRadiomics was used for feature extraction. The binwidth parameter for the feature extraction pipeline was set to 30, the normalization parameter was set to false, and the rest of the parameters were used with default settings. Features were extracted from the original as well as wavelet-filtered images. Extracted features include First Order Statistics (FO), Gray Level Co-occurrence Matrix (GLCM), and Gray Level Run Length Matrix (GLRLM) features, wavelet features, and 3D shape features.

### Feature Selection and Classification

Feature reduction: ComBat is a popular feature harmonization method, which was designed for genomic data,^20^ this method has been well adapted for neuroimaging data through the R package “neuroCombat”^21–23^ and Python package (https://github.com/Jfortin1/neuroCombat). This study utilizes the Python-based NeuroCombat package, it adjusts the extracted feature values to remove scanner or batch effect so that the entire dataset appears as if all images were acquired from the same scanner. It estimates expected values using a linear model that includes biological variables along with additive and multiplicative scanner effects as predictors.^22^ Dataset was harmonized using neurocombat pipeline with default parameters followed by splitting into training (75%) and internal validation dataset (25%) while stratifying for the target variable (H3K27M altered/wild type) to maintain the similar target ratio in both training and testing dataset.

Careful feature selection is important for radiomics-based ML models as radiomics features show a high correlation with one another due to redundancy in the extracted features, this may lead to underfitting or overfitting and hence resulting in poor generalizability of the model. The feature reduction approach was applied to the training dataset to identify the optimal features for the classification. A variance threshold (0.01) was applied to identify the quasi-constant features and similar features in most observations in the dataset were dropped. Features were then dropped based on a correlation greater than 0.9 (strong correlations). The training dataset was then subjected to an adaptive synthesis (ADAsyn) approach for over-sampling the minority class minority class to address the class imbalance. ADAsyn focuses on creating more synthetic samples for minority class instances/samples that are harder to classify, and these are identified through a weighted distribution based on their learning difficulty. By doing so, ADAsyn aims to reduce the bias introduced by data imbalance and adaptively adjust the classification decision boundary towards the more challenging instances/samples, ultimately improving the model’s ability to learn and generalize from the minority class data.^24^ This was done to balance the training dataset for proper ML algorithm training. Training data was scaled/ normalized using the standard scaler module of the scikit learn library, the same transform was then applied to the test dataset. The optimal features were finally selected from the training dataset by the forward selection method of the sequential selector (SFS) module of Python (https://scikit-learn.org/stable/modules/generated/sklearn.feature_selection.SequentialFeatureSelector.html). The Sequential forward selection method is a wrapper method that starts with an empty feature set and incrementally adds features to the set based on the cross-validation score of an estimator to build a single optimal feature set.

Classifiers including the K-nearest neighbor classifier (KNN), decision tree classifier (DTC), and random forest classifier (RFC) were selected based on 5-fold cross-validation and the hyperparameters of the classifiers were tuned using grid search cross-validation method. Hyperparameters of DTC include maximum depth, maximum features, minimum samples split, and minimum samples leaf. The hyperparameters of RFC include bootstrap, criterion, maximum features, minimum samples split, and number of estimators. The hyperparameters for KNN include the number of neighbors and weight. The tuned models were trained on the training dataset and the performance was assessed on the internal and external test datasets. Performance matrices including accuracy (acc), F1-score, precision, recall, and area under the receiver operating characteristics curve (auc) were calculated for individual classes as well as for overall performance. For the overall performance metrices, both macro average and weighted average scores were calculated to address the class imbalance in the test datasets. Performance matrices including accuracy (acc), F1-score, area under the receiver operating characteristics curve (auc), Brier score, sensitivity, specificity, precision, negative predictive value, false positive rate, false negative rate, Matthew’s correlation coefficient (MCC), Youden’s J statistic, and markedness were calculated. For the overall performance metrices macro average and weighted average scores were also calculated to address the class imbalance in the test datasets. ROC-AUC curve, recall-precision curve, calibration, and confusion matrix were calculated for the classifiers. The confidence interval for the AUC scores was calculated using the bootstrap method. The brier score was calculated for the classifiers’ performance using Sklearn’s brier score loss module.

## Results

### Patient Characteristics

The internal study cohort included a total of 124 (63 males/61 females) subjects with midline gliomas consisting of 69 subjects with H3K27M alteration and 55 H3K27M wild-type cases. 37 out of 124 cases were pediatric with 27 of them harboring H3K27M alteration. The mean age of the pediatric group was 8.50 ± 4.81 years for the wild-type group and 10.66 ± 3.72 years for the altered group (Table 1). The remaining 87 cases were adult, with a mean age of 40.3 ± 15.18 years for the H3K27M wild-type group and 33.04 ± 13.04 years for the H3K27M altered group (Table 1). 42 adult cases out of 87 were harboring H3K27M alteration. There was equal distribution between wild type and altered group overall, however number of pediatric cases in the wild-type group was much lower than the number of pediatric cases in the altered group.

**Table 1.** Subject Demographics for the Internal and the External Dataset

Patient demographics:internal dataset						
	H3K27M wild type			H3K27M altered		
	*Adult*	*Pediatric*	*Total*	*Adult*	*Pediatric*	*Total*
Mean age ± standard deviation (years)	40.3 ± 15.18	8.50 ± 4.81	34.21 ± 18.70	33.04 ± 13.04	10.66 ± 3.72	24.28 ± 15.13
Median age (years)	38.5	8.5	34.5	29.0	10.0	21.0
Males (numbers)	24	7	31	22	10	32
Females (numbers)	21	3	24	20	17	37
Patient demographics:external data						
	H3K27M wild type			H3K27M altered		
	*Adult*	Pediatric	Total	Adult	Pediatric	Total
Mean age ± standard deviation (years)	39.37 ± 15.02	9.66 ± 4.16	34.68 ± 17.72	30.57 ± 11.25	11.21 ± 4.11	33.00 ± 13.13
Median age (years)	34.0	11.00	32.00	28.00	11.00	19.00
Males (numbers)	9	3	12	12	10	22
Females (numbers)	7	0	7	7	4	11

The external study cohort included a total of 52 (34 males/18 females) subjects with midline gliomas consisting of 33 subjects with H3K27M alteration and 19 H3K27M wild-type cases. 17 out of 52 cases were pediatric with a mean age of 9.66 ± 4.16 years for the wild-type group and 11.21 ± 4.11 years for the altered group (Table 1). The remaining 35 cases were adult, with a mean age of 39.37 ± 15.02 years for the wild-type group and 30.57 ± 11.25 years for the altered group (Table 1). Like the internal dataset, the number of pediatric cases was much lower in the H3K27M wild type than the H3K27M altered group.

### Tumor Location

**Figure 2.**
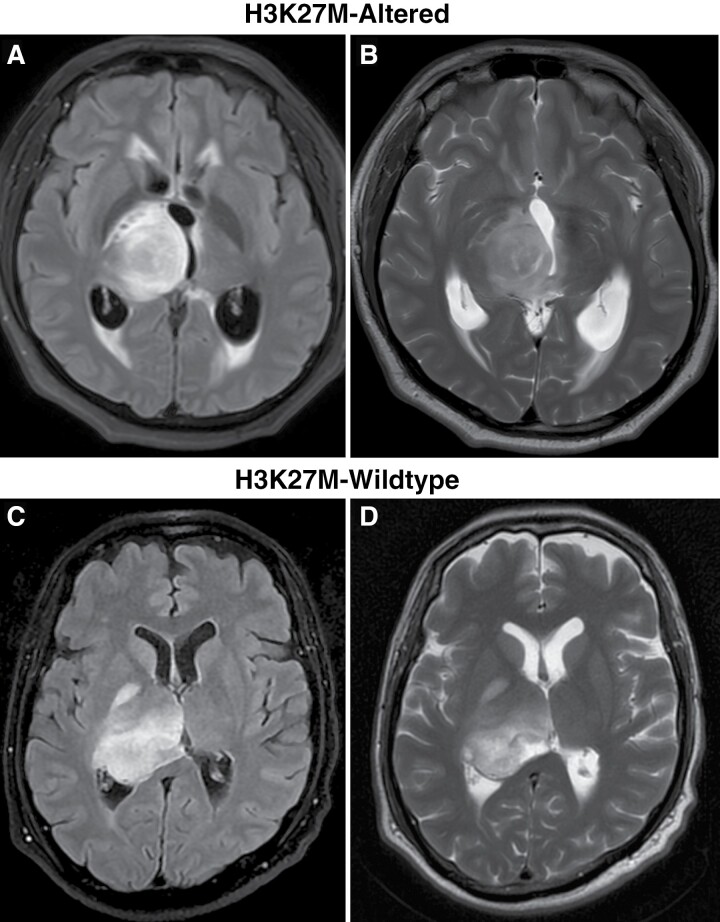
Images for H3K27M-altered and H3K27M-wild-type subjects. (A) T2W-FLAIR image for H3K27M-altered subject. (B) T2W image for H3K27M-altered subject. (C): T2W-FLAIR image for H3K27M-wild-type subject. (D) T2W image for H3K27M-wild-type subject.

Figure 2-a, b depict the T2W-FLAIR and T2W image of a H3K27M-altered subject, while Figure 2-c, d depict the T2W FLAIR and T2W image of a H3K27M-wild-type subject. In the internal study cohort, 54.03% of lesions (*n* = 67) were in the thalamus either involving only the thalamus or extending into adjacent structures. 37.09% (*n* = 46) involved the brainstem, 11.29% involved other midline structures. (Table 2).

**Table 2.** Lesion Location for the Internal Dataset

Lesion location:internal dataset		
Location	Number of subjects (H3K27M Wild type)	Number of subjects (H3K27M altered)
Thalamus	31	36
Brainstem	18	28
Cerebellum	4	1
Posterior third ventricle	1	0
Corpus callosum	0	1
Intraventricular	0	1
Hypothalamus	0	1
Pineal	0	1
Septal	1	0

Among the H3K27M-altered group of 69 subjects, 52.4% of lesions (*n* = 36) were in the thalamus either involving only the thalamus or extending into adjacent structures, 40.57% (*n* = 28) involved the brainstem, 1.4% (*n* = 1) involved each cerebellum, corpus callosum, intraventricular, hypothalamus, and pineal, respectively (Table 2).

In the H3K27M wild-type patients including 55 subjects, 56.36% of lesions (*n* = 31) were in the thalamus either involving only the thalamus or extending into adjacent structures. 32.72% (*n* = 18) involved brainstem, 7.2% (*n* = 4) involved cerebellum, 1.8% (*n* = 1) involved each posterior 3rd ventricle and septal, respectively (Table 2).

### Feature Reduction and Classification

A total of 536 features (Supplementary Table 1) were extracted from the T2W image per subject. There were 18 FO features, 24 GLCM features, 16 GLRLM features, 16 shape features, and 464 wavelet features (a total of 8 decompositions based on the filters and 58 features per decomposition). Three hundred and forty-six features were dropped based on a variance threshold of 0.01. The correlation threshold was then applied to the remaining features, after which the 120 features were dropped from the feature set. The SFS algorithm selected 10 optimal features from the remaining 70 features for further model training. These optimal 10 features include 4 features from the original image and 6 features from the wavelet filter applied image. Original features include FO 90th percentile, FO maximum, GLRLM short run high gray level emphasis, and shape least axis length. The wavelet-filtered image features include FO kurtosis, FO maximum (from 2 different decompositions), FO skewness, GLRLM run entropy, and GLCM autocorrelation. The Correlation matrix of the optimal features is provided in Supplementary Figure 2. The optimal parameters for the 3 classifiers are provided in Supplementary Table 2.

**Figure 3.**
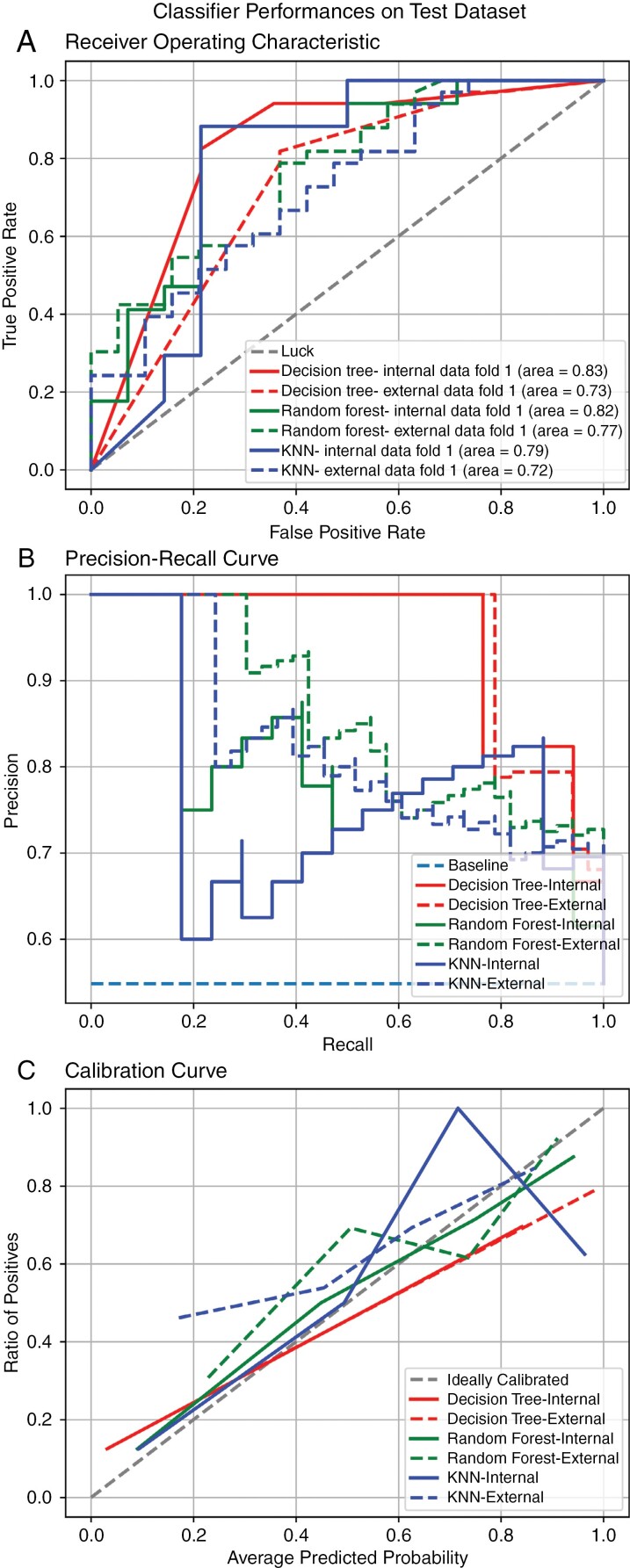
Plots for decision tree classifier, random forest classifier, k-nearest neighbor classifier performances on internal and external test datasets. (A) ROC-AUC curves, (B) Recall-precision curves, and (C) Calibration curves.

The internal test data set had 31 subjects with 14 H3K27M wild type and 17 H3K27M altered cases. The external test dataset had 52 subjects with 19 H3K27M wild type and 33 H3K27M altered cases. Three supervised ML algorithms were trained including DTC, RFC, and KNN. The accuracy, precision, recall, and F1-score of DTC on the internal dataset were 0.80, 0.81,0.81, and 0.81, respectively, and on the external dataset were 0.75, 0.73, 0.72, and 0.73, respectively. Accuracy, precision, recall, and F1 scores for RFC on the internal dataset were 0.806, 0.81, 0.80, and 0.80, respectively and these scores on the external dataset were 0.73, 0.71,0.71, and 0.71, respectively. KNN classifier had the same performance on the internal test dataset as DTC, but KNN had only a 0.615 accuracy score on the external test dataset. Precision, recall, and F1 scores for KNN on the external dataset were 0.65, 0.62, and 0.62, respectively. The validation scores of the DTC, RFC, and KNN on the training dataset were 0.773, 0.807, and 0.787, respectively. The detailed overall performance of the 3 classifiers on both internal and external test datasets is provided in Table 3. The AUC score of DTC, RFC, and KNN on internal test dataset was 0.83 (CI: 0.697–0.947), 0.82 (CI: 0.682–0.943), 0.79 (CI: 0.633–0.929), respectively, and the AUC score of these classifiers on external test dataset was 0.73 (CI: 0.609–0.833), 0.77 (CI: 0.663–0.872), 0.72 (CI: 0.598–0.833), respectively. The ROC-AUC curves for the 3 classifiers on internal and external test datasets are provided in Figure 3-a. The recall-precision curve and calibration curves of the 3 classifiers are provided in Figure 3-b,c, respectively. The confusion matrices of the classifiers are provided in Supplementary Figure 3. The brier scores of DTC, RFC, and KNN on the internal test data are 0.174, 0.177, and 0.184, respectively and the brier scores of these classifiers on the external test data are 0.215, 0.187, and 0.212, respectively.

**Table 3.** Performances of the Three Classifiers

Performance metrics	DT-internal validation	DT- external test	RF-internal validation	RF- external test	KN-internal validation	KN- external test
Accuracy	0.8064 [25/31]	0.75 [39/52]	0.8064 [25/31]	0.7307 [38/52]	0.8064 [25/31]	0.6153 [32/52]
AUC [95% CI]	0.8256 [0.697–0.947]	0.7320 [0.609–0.833]	0.8151 [0.682–0.943]	0.7727 [0.663–0.872]	0.7857 [0.633–0.929]	0.7240 [0.598–0.833]
Brier score	0.174	0.215	0.177	0.187	0.184	0.212
Sensitivity (TPR)	0.8235 [14/17]	0.8181 [27/33]	0.88235 [15/17]	0.7878 [26/33]	0.8823 [15/17]	0.6060 [20/33]
Weighted sensitivity	0.8064 [(0.8235*17+0.7857*14)/31]	0.75 [0.8181*33+0.6315*19]/52	0.8064 [(0.8823*17+0.7142*14)/31]	0.7307 [(0.7878*33+0.6315*19)/52]	0.8064 [(0.8823*17+0.7142*14)/31]	0.6153 [(0.6060*33+0.6315*19)/52]
Macro sensitivity	0.8046 [(0.8235+0.7857)/2]	0.7248 [0.8181+0.6315]/2	0.7983 [(0.8823+0.7142)/2]	0.7097 [(0.7878+0.6315)/2]	0.7983 [(0.8823+0.7142)/2]	0.6188 [(0.6060+0.6315)/2]
Specificity (TNR)	0.7857 [11/14]	0.6315 [12/19]	0.7142 [10/14]	0.6315 [12/19]	0.7142 [10/14]	0.6315 [12/19]
Weighted specificity	0.8027 [(0.7857*17+0.8235*14)/31]	0.6997 [(0.6315*33+0.8181*19)/52]	0.7901 [(0.7142*17+0.8823*14)/31]	0.6886 [(0.6315*33+0.7878*19)/52]	0.7901 [(0.7142*17+0.8823*14)/31]	0.6222 [(0.6315*33+0.6060*19)/52]
Macro specificity	0.8046 [(0.7857+0.8235)/2]	0.7248 [(0.6315+0.8181)/2]	0.7983 [(0.7142+0.8823)/2]	0.7097 [(0.6315+0.7878)/2]	0.7983 [(0.7142+0.8823)/2]	0.6188 [(0.6315+0.6060)/2]
Precision (PPV)	0.8235 [14/17]	0.79411 [27/34]	0.7894 [15/19]	0.7878 [26/33]	0.7894 [15/19]	0.7407 [20/27]
Weighted precision	0.8064 [(0.8235*17+0.7857*14)/31]	0.7475 [(0.79411*33+0.6666*19)/52]	0.8092 [(0.7894*17+0.8333*14)/31]	0.7307 [(0.7878*33+0.6315*19)/52]	0.8092 (0.7894*17+0.8333*14)/31	0.6454 [(0.7407*33+0.48*19)/52]
Macro precision	0.8046 (0.8235+0.7857)/2	0.7303 [(0.7941+0.6666)/2]	0.8114 [(0.7894+0.8333)/2]	0.7097 [(0.7878+0.6315)/2]	0.8114 [(0.7894+0.8333)/2]	0.6103 [(0.7407+0.48)/2]
NPV	0.7857 (11/14)	0.6666 [12/18]	0.8333 [10/12]	0.6315 [12/19]	0.8333 [10/12]	12/25 = 0.48
Weighted NPV	0.8027 (0.7857*17+0.8235*14)/31	0.7132 [(0.6666*33+0.7941*19)/52]	0.8135 [(0.8333*17+0.7894*14)/31]	0.6886 [(0.6315*33+0.7878*19)/52]	0.8135 [(0.8333*17+0.7894*14)/31]	0.5752 [(0.48*33+0.7407*19)/52]
Macro NPV	0.8046 (0.7857+0.8235)/2	0.7303 [(0.6666+0.7941)/2]	0.8114 [(0.8333+0.7894)/2]	0.7097 [(0.6315+0.7878)/2]	0.8114 [(0.8333+0.7894)/2]	0.6103 [(0.48+0.7407)/2]
FPR	0.2142 (3/14)	0.3684 [7/19]	0.2857 [4/14]	0.3684 [7/19]	0.2857 [4/14]	0.3684 [7/19]
Weighted FPR	0.1972 (0.2142*17+0.1764*14)/31	0.3002 [(0.3684*33+0.1818*19)/52]	0.2098 [(0.2857*17+0.1176*14)/31]	0.3113 [(0.3684*33+0.2121*19)/52]	0.2098 [(0.2857*17+0.1176*14)/31]	0.3777 [(0.3684*33+0.3939* 19)/52]
Macro FPR	0.1953 (0.2142+0.1764)/2	0.2751 [(0.3684+0.1818)/2]	0.2016 [(0.2857+0.1176)/2]	0.2902 [(0.3684+0.2121)/2]	0.2016 [(0.2857+0.1176)/2]	0.3811 [(0.3684+0.3939)/2]
FNR	0.1764 (3/17)	0.1818 [6/33]	0.1176 [2/17]	0.2121 [7/33]	0.1176 [2/17]	0.3939 [13/33]
Weighted FNR	0.1935 (0.1764*17+0.2142*14)/31	0.25 [(0.1818*33+0.3684*19)/52]	0.1935 [(0.11764*17+0.2857*14)/31]	0.2692 [(0.2121*33+0.3684*19)/52]	0.1935 [(0.1176*17+0.2857 *14)/31]	0.3846 [(0.3939*33+0.3684* 19)/52]
Macro FNR	0.1953 (0.1764+0.2142)/2	0.2751 [(0.1818+0.3684)/2]	0.2016 [(0.11764+0.2857)/2]	0.2902 [(0.2121+0.3684)/2]	0.2016 [(0.1176+0.2857)/2]	0.3811 [(0.3939+0.3684)/2]
F1-score	0.8235 (28/34)	0.8059 [54/67]	0.8333 [30/36]	0.7878 [52/66]	0.8333 [30/36]	0.6666 [40/60]
Weighted F1-score	0.8064 (0.8235*17+0.7857*14)/31	0.7484 [(0.8059*33+0.6486*19)/52]	0.8043 [(0.8333*17+0.7692*14)/31]	0.7307 (0.7878*33+0.6315*19)/52	0.8043 [(0.8333*17+0.7692*14)/31]	0.6223 [(0.6666*33+0.5454*19)/52]
Macro F1-score	0.8046 (0.8235+0.7857)/2	0.7273 [(0.8059+0.6486)/2]	0.8012 [(0.8333+0.7692)/2]	0.7097 [(0.7878+0.6315)/2]	0.8012 [(0.8333+0.7692)/2]	0.6060 [(0.6666+0.5454)/2]
MCC	0.609	0.4552	0.6095	0.4194	0.6095	0.2290
Youden’s J statistic	0.609	0.4497	0.5966	0.4194	0.5966	0.2376
Markedness	0.609	0.4607	0.6228	0.4194	0.6228	0.2207

The values in the gray color within the square brackets are the numerosity for the respective metrics depicting how the value was calculated using the confusion metrics. The macro-averaged metrics represent the average of respective metrics calculated for each class separately. The weighted averaged metric represents the weighted sum of the metrics where each class’s metric is weighted by the proportion of instances it represents.

CI, confidence interval; TPR, true positive rate; TNR, true negative rate; PPV, positive predictive value; NPV, negative predictive value; FPR, false positive rate; FNR, false negative rate; MCC, Matthew’s correlation coefficient.

## Discussion

In this study, ML-based models were trained using radiomics features extracted on conventional T2W images original and wavelet filtered. The feature set was subjected to a careful feature reduction approach to get 10 optimal features for the classification task. In addition, the models’ performances were also carefully assessed on both internal as well as external test data. The models’ performances were evaluated using macro averaged and weighted averaged recall, precision, and F1 score metrices to address the class imbalance in the test datasets. Recall-precision curves, ROC-AUC curves, confidence interval of AUC scores, and calibration curves for the models were calculated. Brier scores were calculated for the classifiers. It measures the mean squared difference between the predicted probability and the actual outcomes. Brier scores of all the classifiers were less than 0.22 for both internal and external datasets. DTC, RFC, and KNN, all achieved an accuracy of approximately 80% on the internal dataset. These classifiers achieved accuracy of 75%, 73%, and 61.5%, respectively, on the external dataset. The reason for this drop in accuracy might be that the features of the external test dataset come from different data distributions. The H3K27M altered class contains the same tumor class, ie, DMG while the wild-type class contains different tumor entities including astrocytoma, glioblastoma, etc. This results in heterogeneous features within the wild-type class. Therefore, the availability of a sufficient sample size of H3K27M wild type is necessary to capture this heterogeneity. Hence, another potential reason for the drop in accuracy might be more mistakes made by the classifiers in the H3K27M wild-type class in both internal and external test datasets. Overall, the DTC and RFC achieved decent accuracies on the external dataset.

In this study, we used T2W images on account of the availability of good-quality images in the maximal number of cases with consistency of sequence parameters. We targeted a single sequence because the inclusion of multiple sequences would have further reduced the sample size due to the non-availability or poor quality of some of the acquisitions. T2W images are a useful sequence for delineating the extent of tumor and tumoral edema. Furthermore, we believed that tumor segmentation was more feasible on T2W images compared to the contrast-enhanced images pertaining to the heterogenous contrast enhancement pattern of DMGs and poor visualization of surrounding edema.

H3K27 methylation is known to be a hallmark of gene repression. The H3K27M alteration has significant implications for both pediatric and adult patients in terms of tumor behavior, treatment options, and prognosis.^25^ In the pediatric population this alteration is frequently observed in a defining molecular marker. These tumors typically arise in the pons region and are highly aggressive making surgical resection challenging. In adults, these alterations can occur at various locations such as the thalamus, spinal cord, and other midline structures and confer a bad prognosis.^3,26,27^ H3K27M alteration inhibits the activity of the polycomb repressive complex 2, which results in widespread changes in the gene expression contributing to tumor development and progression.^28^ Prediction of H3K27M alteration is of prime importance in case of inconclusive biopsy or in the absence of it. Noninvasive differentiation between H3K27M-altered and wild-type tumors using semantic features such as tumor size, enhancement, internal necrotic changes, or infiltrative appearance, is difficult because of the absence of significant differences between these features.^12,13^

The accuracy scores of our model based on T2 weighted images were in line with the previous studies conducted on single as well as multiple sequences. Wu et al. developed a nomogram based on multi-modalities on 107 pediatric patients and achieved an AUC of 0.92.^29^ Zhuo et al.^30^ reported accuracy of 88% and 86% on test data set and an independent validation cohort using radiomics features of amide proton transfer (APT) weighted images; however, APT sequences are not available at all medical centers which restrict the utility of this sequence. Su et al.^31^ using a cohort of 100 subjects, optimized an automated Tree-based Pipeline Optimization Tool (TPOT) on FLAIR radiomics features and achieved accuracy between 0.60 and 0.84 on the test dataset. Chang et al.,^26^ trained RFC on features extracted from multiple MR modalities of 151 subjects, which yielded an accuracy score of 0.844. However, to address the class imbalance, data from the minority class was duplicated in the training dataset. In our study, we used the Adaptive synthesis technique to oversample the minority class in the training dataset, which is an improved technique for dealing with class imbalance. Khalid et al.^32^ performed a multiclass classification conducted radiomics study using multiple modalities to predict H3.1, TP53, and ACVR1 on a cohort of 80 subjects and achieved an accuracy of 0.878. In this study, 63 out of 80 subjects had the H3K27M alteration status, out of which only 4 were WT and the rest were altered. Kandemirli et al^33^ trained XGBoost on radiomics features extracted from multi-modal MR images of 109 subjects, from 3 academic centers and achieved an accuracy of 72.7% on the test dataset. Guo et al.,^34^ in a cohort of 102 subjects, trained multiple ML models on radiomics features extracted from multiple MR modalities and achieved an AUC of 0.969, and an accuracy of 0.767. However, there was a high-class imbalance in the cohort with only 27 H3K27M altered cases, this probably led to a low sensitivity of 0.125 on the test dataset as stated by the authors as one of the limitations in their study. Li et al.^35^ found a high overlap between imaging features of H3K27M altered and wild-type tumors which included radiomics and visibly accessible features. This study, however, was conducted on a limited sample size of 30 subjects. A meta-analysis by Hua et al.^19^ of 7 studies from 2019 to 2021, reported the pooled sensitivity of H3K27M prediction was 0.78 (95% CI: 0.66–0.87), and a pooled specificity of 0.85 (95% CI: 0.76–0.91). One study included a single MR modality, and the rest of the studies were conducted on multimodal data including advanced MRI techniques such as APT and ASL. The meta-analysis suggested that combining conventional MR imaging such as T1-weighted, T2-weighted, ADC and advanced MR imaging such as APT weighted may achieve a promising performance of H3 K27M-mutant prediction with a high sensitivity and specificity. However, these studies had a major class imbalance in the respective datasets.

There are a few other predictive studies for H3K27M alteration that do not involve radiomics features but other semi-quantitative and qualitative features. Chen et al.^27^ trained a logistic regression model on quantitative features of apparent diffusion coefficient (ADC) maps from a small cohort of 38 patients, and achieved an AUC score of 0.872 in defining the H3K27M alteration status. Jo et al.^3^ trained a random forest (RF) classifier on clinical and qualitative, semi-quantitative radiological features from 41 spinal cord glioma patients that yielded an accuracy of 0.634. Piccardo et al.,^36^ did ROC analysis over 18F-DOPA Tumor/Striatum (T/S) ratios from 22 pediatric patients and achieved an AUC score of 0.94. Authors have also investigated other parameters such as relative minimum ADC, 1H-MRS, relative arterial spin labeling-derived cerebral blood flow, and 18F-DOPA uptake Tumor/Normal tissue ratios. This study was carried out on a small dataset, including modalities that are difficult to post-process and are not available at all medical centers. Raab et al.^37^ found that skewness and kurtosis derived from ADC maps of 57 subjects differ significantly between the H3K27M altered and midline glioblastoma cohort. Chauhan et al.,^38^ in a cohort of 123 subjects assessed the Visually AcceSAble Rembrandt Images feature, Intra Tumoral Susceptibility Signal score from multiple MR modalities and Kathrani et al.^39^ used diffusion, perfusion parameters of 94 subjects to differentiate between H3K27M altered and wild-type cases. All the previously reported radiomics studies except Kandemirli et al.,^33^ either included only the pediatric dataset or only the adult dataset and therefore cannot be compared directly with our study.

From the methodological point of view, this study follows important steps for in radiomics study in oncology,^40^ including preprocessing of the images for normalization and removal of artifacts, resampling the images to 1 × 1 × 1 mm^3^ voxel size, reporting the parameters used while extracting the features and model training, and feature harmonization. To account for class imbalance and a relatively small data size, yet comparable to previous studies, we have used the ADAsyn algorithm only on the training dataset to oversample the minority class and hence increase the size of the training dataset. Rather than creating duplicates, it adaptively generates data samples according to the data distributions of the minority class. More synthetic data is generated for minority class samples that are difficult to learn compared to those minority samples that are comparatively easier to learn.

Our study has several limitations. Even though the size of our dataset is comparable to previous studies, it is still relatively small. Increasing the sample size can also help in addressing the data mismatch that can occur due to different data distributions in the test dataset. Another limitation of this study is that the lesions were segmented semi-automatically which can lead to some bias, given the infiltrative nature of the tumors. The models couldn’t be tested on any open-source data set due to the unavailability of the open-source data for H3K27M alteration. Although this mutation can occur in children as well as young adults and the features were selected to represent the complete cohort, this study does not consider the variability in the radiomics features that might occur between the pediatric and adult features. Finally, several works remain to be accomplished in the future. The use of a multimodal approach on a larger dataset can further help in training and tuning a better model. The use of fully automated algorithms for lesion segmentation can minimize human interference and promote an automated pipeline for such predictive models. A subgroup analysis of pediatric and adult populations could provide more insight into the radiomic feature differences between these subsets. A more general problem with the radiomics approach is the lack of biological interpretability of these features.

In conclusion, Radiomics features extracted from conventional T2W images can be used to predict H3K27M alteration in DMGs. DTC and RFC models trained in this study are sensitive towards identifying the altered group and could identify the alteration with a good recall score in the test dataset. Further studies in this direction can help build a noninvasive tool to support decision-making for the diagnosis of DMGs.

## Supplementary Material

vdae153_suppl_Supplementary_Material
