# Supplementary material for: Prediction of H3K27M alteration in midline gliomas of the brain using radiomics: A multi-institute study
Source: Neurooncol Adv. 2024 Sep 10;6(1):vdae153. doi: 10.1093/noajnl/vdae153 (PMC11600333; doi:10.1093/noajnl/vdae153)
Supplement: vdae153_suppl_Supplementary_Material [file vdae153_suppl_supplementary_material.docx]

***Prediction of H3K27M alteration in midline gliomas of brain using radiomics- A multi-institute study.***

| Original features no filters | wavelet-HHH filtered features | wavelet-HHL filtered features | wavelet-HLH filtered features | wavelet-HLL filtered features |
| --- | --- | --- | --- | --- |
| firstorder 90Percentile | firstorder 90Percentile | firstorder 90Percentile | firstorder 90Percentile | firstorder 90Percentile |
| firstorder Energy | firstorder Energy | firstorder Energy | firstorder Energy | firstorder Energy |
| firstorder Entropy | firstorder Entropy | firstorder Entropy | firstorder Entropy | firstorder Entropy |
| Firstorder Interquartile Range | firstorder Interquartile Range | firstorder Interquartile Range | firstorder Interquartile Range | firstorder Interquartile Range |
| firstorder Kurtosis | firstorder Kurtosis | firstorder Kurtosis | firstorder Kurtosis | firstorder Kurtosis |
| firstorder Maximum | firstorder Maximum | firstorder Maximum | firstorder Maximum | firstorder Maximum |
| firstorder Mean | firstorder Mean | firstorder Mean | firstorder Mean | firstorder Mean |
| firstorder Mean Absolute Deviation | firstorder Mean Absolute Deviation | firstorder Mean Absolute Deviation | firstorder Mean Absolute Deviation | firstorder Mean Absolute Deviation |
| firstorder Median | firstorder Median | firstorder Median | firstorder Median | firstorder Median |
| firstorder Minimum | firstorder Minimum | firstorder Minimum | firstorder Minimum | firstorder Minimum |
| firstorder Range | firstorder Range | firstorder Range | firstorder Range | firstorder Range |
| firstorder Robust Mean Absolute Deviation | firstorder Robust Mean Absolute Deviation | firstorder Robust Mean Absolute Deviation | firstorder Robust Mean Absolute Deviation | firstorder Robust Mean Absolute Deviation |
| Firstorder Root Mean Squared | firstorder Root Mean Squared | firstorder Root Mean Squared | firstorder Root Mean Squared | firstorder Root Mean Squared |
| firstorder Skewness | firstorder Skewness | firstorder Skewness | firstorder Skewness | firstorder Skewness |
| firstorder Total Energy | firstorder Total Energy | firstorder Total Energy | firstorder Total Energy | firstorder Total Energy |
| firstorder Uniformity | firstorder Uniformity | firstorder Uniformity | firstorder Uniformity | firstorder Uniformity |
| firstorder Variance | firstorder Variance | firstorder Variance | firstorder Variance | firstorder Variance |
| glcm Autocorrelation | glcm Autocorrelation | glcm Autocorrelation | glcm Autocorrelation | glcm Autocorrelation |
| glcm Cluster Prominence | glcm Cluster Prominence | glcm Cluster Prominence | glcm Cluster Prominence | glcm Cluster Prominence |
| glcm Cluster Shade | glcm Cluster Shade | glcm Cluster Shade | glcm Cluster Shade | glcm Cluster Shade |
| glcm Cluster Tendency | glcm Cluster Tendency | glcm Cluster Tendency | glcm Cluster Tendency | glcm Cluster Tendency |
| glcm Contrast | glcm Contrast | glcm Contrast | glcm Contrast | glcm Contrast |
| glcm Correlation | glcm Correlation | glcm Correlation | glcm Correlation | glcm Correlation |
| glcm Difference Average | glcm Difference Average | glcm Difference Average | glcm Difference Average | glcm Difference Average |
| glcm Difference Entropy | glcm Difference Entropy | glcm Difference Entropy | glcm Difference Entropy | glcm Difference Entropy |
| glcm Difference Variance | glcm Difference Variance | glcm Difference Variance | glcm Difference Variance | glcm Difference Variance |
| glcm Id | glcm Id | glcm Id | glcm Id | glcm Id |
| glcm Idm | glcm Idm | glcm Idm | glcm Idm | glcm Idm |
| glcm Idmn | glcm Idmn | glcm Idmn | glcm Idmn | glcm Idmn |
| glcm Idn | glcm Idn | glcm Idn | glcm Idn | glcm Idn |
| glcm Imc1 | glcm Imc1 | glcm Imc1 | glcm Imc1 | glcm Imc1 |
| glcm Imc2 | glcm Imc2 | glcm Imc2 | glcm Imc2 | glcm Imc2 |
| glcm Inverse Variance | glcm Inverse Variance | glcm Inverse Variance | glcm Inverse Variance | glcm Inverse Variance |
| glcm Joint Average | glcm Joint Average | glcm Joint Average | glcm Joint Average | glcm Joint Average |
| glcm Joint Energy | glcm Joint Energy | glcm Joint Energy | glcm Joint Energy | glcm Joint Energy |
| glcm Joint Entropy | glcm Joint Entropy | glcm Joint Entropy | glcm Joint Entropy | glcm Joint Entropy |
| glcm Maximum Probability | glcm Maximum Probability | glcm Maximum Probability | glcm Maximum Probability | glcm Maximum Probability |
| glcm MCC | glcm MCC | glcm MCC | glcm MCC | glcm MCC |
| glcm Sum Average | glcm Sum Average | glcm Sum Average | glcm Sum Average | glcm Sum Average |
| glcm Sum Entropy | glcm Sum Entropy | glcm Sum Entropy | glcm Sum Entropy | glcm Sum Entropy |
| glcm Sum Squares | glcm Sum Squares | glcm Sum Squares | glcm Sum Squares | glcm Sum Squares |
| Glrlm Gray Level Non-Uniformity | Glrlm Gray Level Non-Uniformity | glrlm Gray Level Non-Uniformity | glrlm Gray Level Non-Uniformity | Glrlm Gray Level Non-Uniformity |
| glrlm Gray Level Non-Uniformity Normalized | glrlm Gray Level Non-Uniformity Normalized | glrlm Gray Level Non-Uniformity Normalized | glrlm Gray Level Non-Uniformity Normalized | glrlm Gray Level Non-Uniformity Normalized |
| glrlm Gray Level Variance | glrlm Gray Level Variance | glrlm Gray Level Variance | glrlm Gray Level Variance | glrlm Gray Level Variance |
| glrlm High Gray Level Run Emphasis | glrlm High Gray Level Run Emphasis | glrlm High Gray Level Run Emphasis | glrlm High Gray Level Run Emphasis | glrlm High Gray Level Run Emphasis |
| glrlm Long Run Emphasis | glrlm Long Run Emphasis | glrlm Long Run Emphasis | glrlm Long Run Emphasis | glrlm Long Run Emphasis |
| glrlm Long Run High Gray Level Emphasis | glrlm Long Run High Gray Level Emphasis | glrlm Long Run High Gray Level Emphasis | glrlm Long Run High Gray Level Emphasis | glrlm Long Run High Gray Level Emphasis |
| glrlm Long Run Low Gray Level Emphasis | glrlm Long Run Low Gray Level Emphasis | glrlm Long Run Low Gray Level Emphasis | glrlm Long Run Low Gray Level Emphasis | glrlm Long Run Low Gray Level Emphasis |
| glrlm Low Gray Level Run Emphasis | glrlm Low Gray Level Run Emphasis | glrlm Low Gray Level Run Emphasis | glrlm Low Gray Level Run Emphasis | glrlm Low Gray Level Run Emphasis |
| glrlm Run Entropy | glrlm Run Entropy | glrlm Run Entropy | glrlm Run Entropy | glrlm Run Entropy |
| glrlm Run Length Non-Uniformity | glrlm Run Length Non-Uniformity | glrlm Run Length Non-Uniformity | glrlm Run Length Non-Uniformity | glrlm Run Length Non-Uniformity |
| glrlm Run Length Non-Uniformity Normalized | glrlm Run Length Non-Uniformity Normalized | glrlm Run Length Non-Uniformity Normalized | glrlm Run Length Non-Uniformity Normalized | glrlm Run Length Non-Uniformity Normalized |
| glrlm Run Percentage | glrlm Run Percentage | glrlm Run Percentage | glrlm Run Percentage | glrlm Run Percentage |
| glrlm Run Variance | glrlm Run Variance | glrlm Run Variance | glrlm Run Variance | glrlm Run Variance |
| glrlm Short Run Emphasis | glrlm Short Run Emphasis | glrlm Short Run Emphasis | glrlm Short Run Emphasis | glrlm Short Run Emphasis |
| glrlm Short Run High Gray Level Emphasis | glrlm Short Run High Gray Level Emphasis | glrlm Short Run High Gray Level Emphasis | glrlm Short Run High Gray Level Emphasis | glrlm Short Run High Gray Level Emphasis |
| glrlm Short Run Low Gray Level Emphasis | glrlm Short Run Low Gray Level Emphasis | glrlm Short Run Low Gray Level Emphasis | glrlm Short Run Low Gray Level Emphasis | glrlm Short Run Low Gray Level Emphasis |
| firstorder 10 Percentile | firstorder 10 Percentile | firstorder 10 Percentile | firstorder 10 Percentile | firstorder 10 Percentile |
|  |  |  |  |  |
| wavelet-LHH filtered features | **wavelet-LHL filtered features** | **wavelet-LLH filtered features** | **wavelet-LLL filtered features** | **shape features** |
| firstorder 90Percentile | firstorder 90Percentile | firstorder 90Percentile | firstorder 90Percentile | Elongation |
| firstorder Energy | firstorder Energy | firstorder Energy | firstorder Energy | Flatness |
| firstorder Entropy | firstorder Entropy | firstorder Entropy | firstorder Entropy | Least Axis Length |
| firstorder Interquartile Range | firstorder Interquartile Range | firstorder Interquartile Range | firstorder Interquartile Range | Major Axis Length |
| firstorder Kurtosis | firstorder Kurtosis | firstorder Kurtosis | firstorder Kurtosis | Maximum2DDiameterColumn |
| firstorder Maximum | firstorder Maximum | firstorder Maximum | firstorder Maximum | Maximum2DDiameterRow |
| firstorder Mean | firstorder Mean | firstorder Mean | firstorder Mean | Maximum2DDiameterSlice |
| firstorder Mean Absolute Deviation | firstorder Mean Absolute Deviation | firstorder Mean Absolute Deviation | firstorder Mean Absolute Deviation | Maximum3DDiameter |
| firstorder Median | firstorder Median | firstorder Median | firstorder Median | Mesh Volume |
| firstorder Minimum | firstorder Minimum | firstorder Minimum | firstorder Minimum | Minor Axis Length |
| firstorder Range | firstorder Range | firstorder Range | firstorder Range | Sphericity |
| firstorder Robust Mean Absolute Deviation | firstorder Robust Mean Absolute Deviation | firstorder Robust Mean Absolute Deviation | firstorder Robust Mean Absolute Deviation | Surface Area |
| firstorder Root Mean Squared | firstorder Root Mean Squared | firstorder Root Mean Squared | firstorder Root Mean Squared | Surface Volume Ratio |
| firstorder Skewness | firstorder Skewness | firstorder Skewness | firstorder Skewness | Voxel Volume |
| firstorder Total Energy | firstorder Total Energy | firstorder Total Energy | firstorder Total Energy |  |
| firstorder Uniformity | firstorder Uniformity | firstorder Uniformity | firstorder Uniformity |  |
| firstorder Variance | firstorder Variance | firstorder Variance | firstorder Variance |  |
| glcm Autocorrelation | glcm Autocorrelation | glcm Autocorrelation | glcm Autocorrelation |  |
| glcm Cluster Prominence | glcm Cluster Prominence | glcm Cluster Prominence | glcm Cluster Prominence |  |
| glcm Cluster Shade | glcm Cluster Shade | glcm Cluster Shade | glcm Cluster Shade |  |
| glcm Cluster Tendency | glcm Cluster Tendency | glcm Cluster Tendency | glcm Cluster Tendency |  |
| glcm Contrast | glcm Contrast | glcm Contrast | glcm Contrast |  |
| glcm Correlation | glcm Correlation | glcm Correlation | glcm Correlation |  |
| glcm Difference Average | glcm Difference Average | glcm Difference Average | glcm Difference Average |  |
| glcm Difference Entropy | glcm Difference Entropy | glcm Difference Entropy | glcm Difference Entropy |  |
| glcm Difference Variance | glcm Difference Variance | glcm Difference Variance | glcm Difference Variance |  |
| glcm Id | glcm Id | glcm Id | glcm Id |  |
| glcm Idm | glcm Idm | glcm Idm | glcm Idm |  |
| glcm Idmn | glcm Idmn | glcm Idmn | glcm Idmn |  |
| glcm Idn | glcm Idn | glcm Idn | glcm Idn |  |
| glcm Imc1 | glcm Imc1 | glcm Imc1 | glcm Imc1 |  |
| glcm Imc2 | glcm Imc2 | glcm Imc2 | glcm Imc2 |  |
| glcm Inverse Variance | glcm Inverse Variance | glcm Inverse Variance | glcm Inverse Variance |  |
| glcm Joint Average | glcm Joint Average | glcm Joint Average | glcm Joint Average |  |
| glcm Joint Energy | glcm Joint Energy | glcm Joint Energy | glcm Joint Energy |  |
| glcm Joint Entropy | glcm Joint Entropy | glcm Joint Entropy | glcm Joint Entropy |  |
| glcm Maximum Probability | glcm Maximum Probability | glcm Maximum Probability | glcm Maximum Probability |  |
| glcm MCC | glcm MCC | glcm MCC | glcm MCC |  |
| glcm Sum Average | glcm Sum Average | glcm Sum Average | glcm Sum Average |  |
| glcm Sum Entropy | glcm Sum Entropy | glcm Sum Entropy | glcm Sum Entropy |  |
| glcm Sum Squares | glcm Sum Squares | glcm Sum Squares | glcm Sum Squares |  |
| glrlm Gray Level Non-Uniformity | glrlm Gray Level Non-Uniformity | glrlm Gray Level Non-Uniformity | glrlm Gray Level Non-Uniformity |  |
| glrlm Gray Level Non-Uniformity Normalized | glrlm Gray Level Non-Uniformity Normalized | glrlm Gray Level Non-Uniformity Normalized | glrlm Gray Level Non-Uniformity Normalized |  |
| glrlm Gray Level Variance | glrlm Gray Level Variance | glrlm Gray Level Variance | glrlm Gray Level Variance |  |
| glrlm High Gray Level Run Emphasis | glrlm High Gray Level Run Emphasis | glrlm High Gray Level Run Emphasis | glrlm High Gray Level Run Emphasis |  |
| glrlm Long Run Emphasis | glrlm Long Run Emphasis | glrlm Long Run Emphasis | glrlm Long Run Emphasis |  |
| glrlm Long Run High Gray Level Emphasis | glrlm Long Run High Gray Level Emphasis | glrlm Long Run High Gray Level Emphasis | glrlm Long Run High Gray Level Emphasis |  |
| glrlm Long Run Low Gray Level Emphasis | glrlm Long Run Low Gray Level Emphasis | glrlm Long Run Low Gray Level Emphasis | glrlm Long Run Low Gray Level Emphasis |  |
| glrlm Low Gray Level Run Emphasis | glrlm Low Gray Level Run Emphasis | glrlm Low Gray Level Run Emphasis | glrlm Low Gray Level Run Emphasis |  |
| glrlm Run Entropy | glrlm Run Entropy | glrlm Run Entropy | glrlm Run Entropy |  |
| glrlm Run Length Non-Uniformity | glrlm Run Length Non-Uniformity | glrlm Run Length Non-Uniformity | glrlm Run Length Non-Uniformity |  |
| glrlm Run Length Non-Uniformity Normalized | glrlm Run Length Non-Uniformity Normalized | glrlm Run Length Non-Uniformity Normalized | glrlm Run Length Non-Uniformity Normalized |  |
| glrlm Run Percentage | glrlm Run Percentage | glrlm Run Percentage | glrlm Run Percentage |  |
| glrlm Run Variance | glrlm Run Variance | glrlm Run Variance | glrlm Run Variance |  |
| glrlm Short Run Emphasis | glrlm Short Run Emphasis | glrlm Short Run Emphasis | glrlm Short Run Emphasis |  |
| glrlm Short Run High Gray Level Emphasis | glrlm Short Run High Gray Level Emphasis | glrlm Short Run High Gray Level Emphasis | glrlm Short Run High Gray Level Emphasis |  |
| glrlm Short Run Low Gray Level Emphasis | glrlm Short Run Low Gray Level Emphasis | glrlm Short Run Low Gray Level Emphasis | glrlm Short Run Low Gray Level Emphasis |  |
| firstorder 10 Percentile | firstorder 10 Percentile | firstorder 10 Percentile | firstorder 10 Percentile |  |

Supplementary Table1: List of all the features extracted.

glcm: Gray Level Co-occurrence Matrix; Id: Inverse Difference; Idm: Inverse Difference Moment; Idmn: Inverse Difference Moment Normalized; Idn: Inverse Difference Normalized; Imc1: Informational Measure of Correlation1; Imc2: Informational Measure of Correlation2; MCC: Maximal Correlation Coefficient; Glrlm: Gray Level Run Length Matrix.

| **Decision Tree Classifier** | |
| --- | --- |
| Criterion | Entropy |
| Max Features | 4 |
| Min Samples Leaf | 3 |
| Min Samples Split | 5 |
| Random state | 19 |
| **Random Forest Classifier** | |
| Bootstrap | False |
| Criterion | Entropy |
| Max Features | 7 |
| Min samples split | 3 |
| Estimators | 300 |
| Random state | 2 |
| **k Nearest Neighbour Classifier** | |
| Neighbours | 7 |
| Weight | Distance |

Supplementary Table1: Hyper Parameters of the classifiers


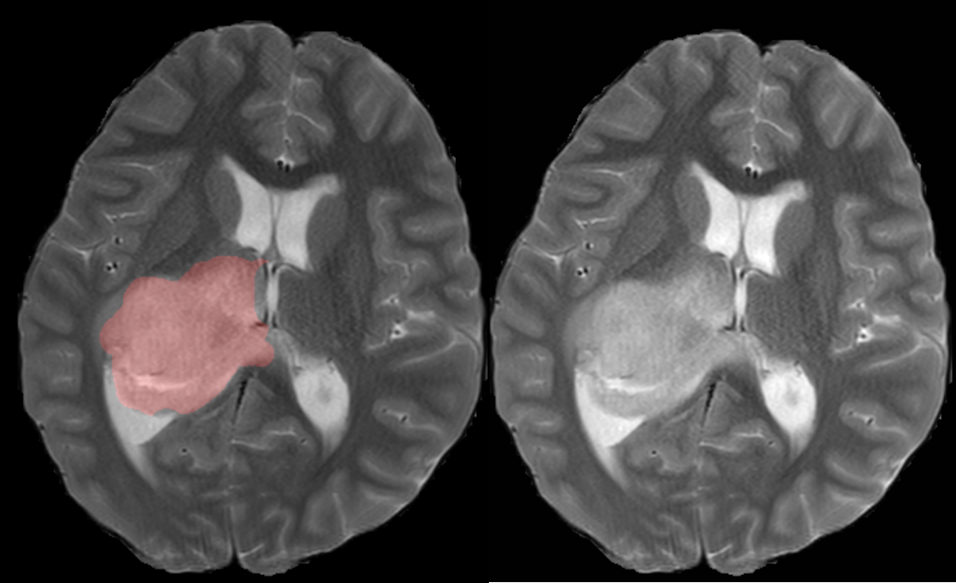


Supplementary Figure1: Segmentation example on T2 weighted image


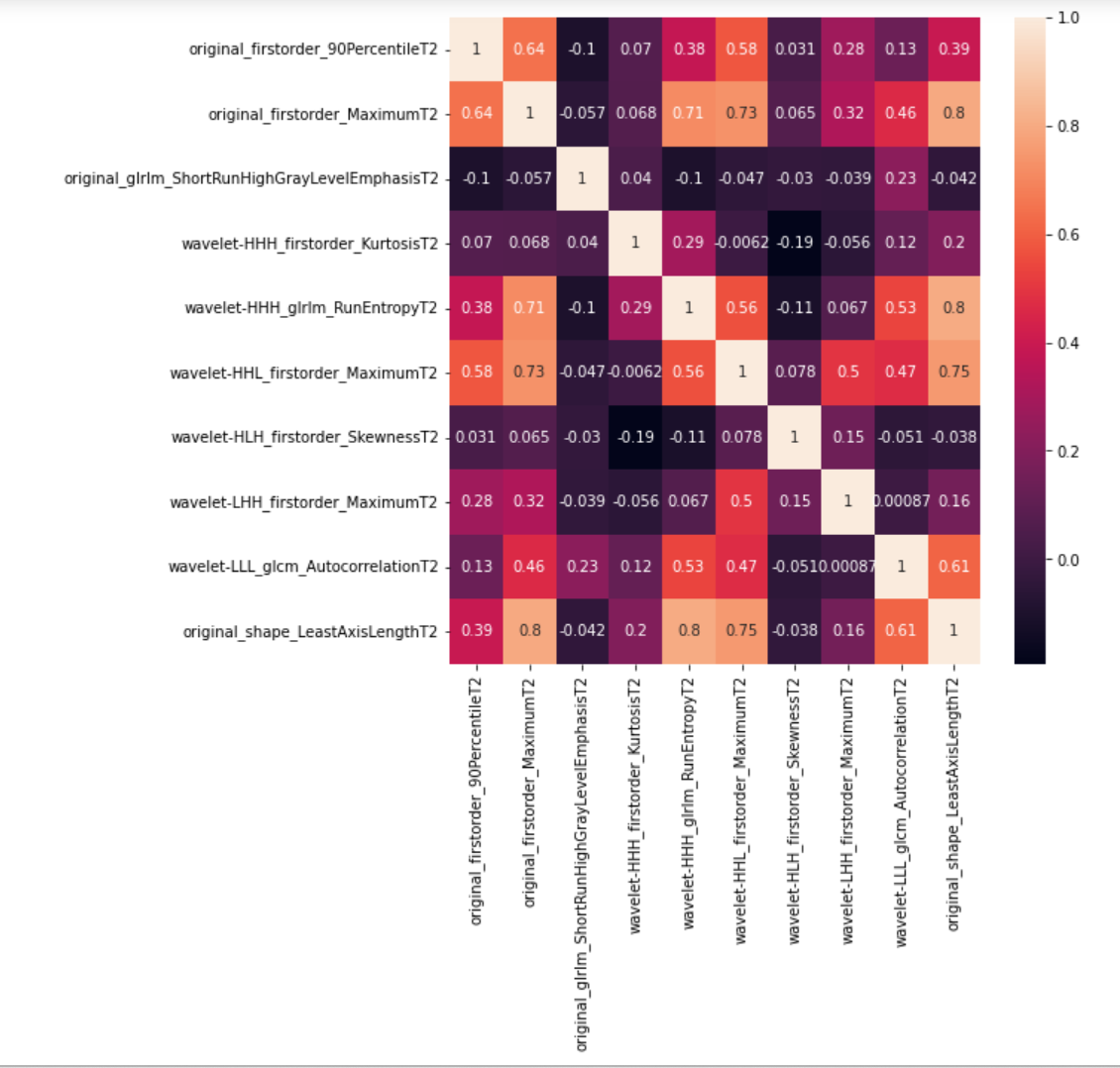


Supplementary Figure2: Heatmap of correlation of the optimal feature set


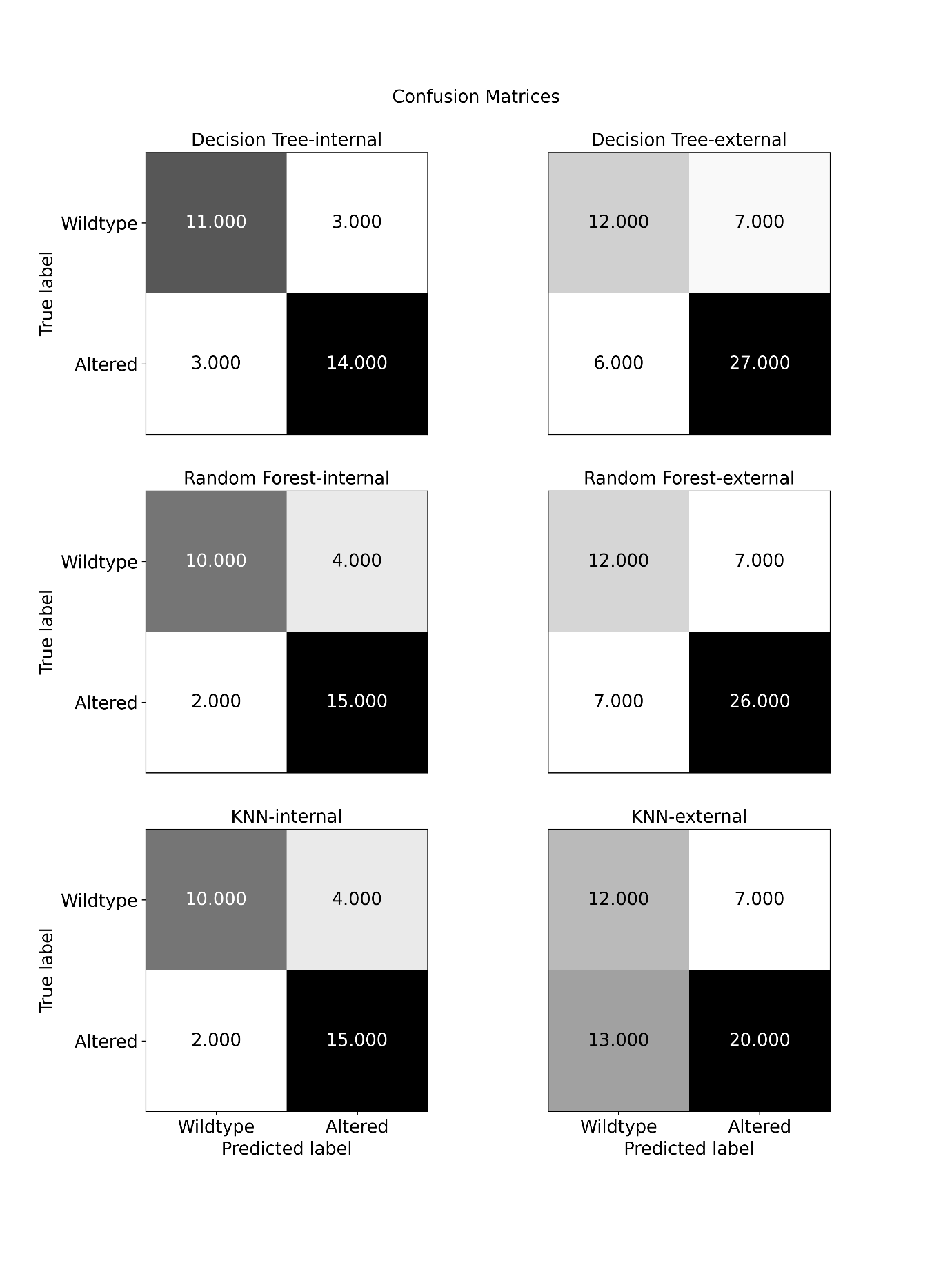


Supplementary Figure3: Confusion matrices of internal and external data set for different classifiers. Decision tree classifier (Top), Random forest classifier (middle), and k- nearest neighbour classifier (bottom).
